# Design and Rationale of the Phase 2 Baricitinib Study in Apolipoprotein L1–Mediated Kidney Disease (JUSTICE)

**DOI:** 10.1016/j.ekir.2024.06.033

**Published:** 2024-06-27

**Authors:** Opeyemi A. Olabisi, Nadine J. Barrett, Anika Lucas, Maurice Smith, Kenisha Bethea, Karen Soldano, Stephanie Croall, Azita Sadeghpour, Hrishikesh Chakraborty, Myles Wolf

**Affiliations:** 1 Division of Nephrology, Duke University School of Medicine, Durham, North Carolina, USA; 2 Duke Molecular Physiology Institute, Duke University School of Medicine, Durham, North Carolina, USA; 3 Atrium Health/Wake Forest Comprehensive Cancer Center and Maya Angelo Center for Health Equity, Wake Forest School of Medicine, Wake Forest, North Carolina, USA; 4 Department of Social Science and Health Policy, Division of Population Health Sciences, Wake Forest School of Medicine, Winston-Salem, North Carolina, USA; 5 Maya Angelo Center for Health Equity, Wake Forest School of Medicine, Winston-Salem, North Carolina, USA; 6 Duke Clinical and Translational Science Institute, Duke University School of Medicine, Durham, North Carolina, USA; 7 Duke Precision Medicine Program, Department of Medicine, Duke University School of Medicine, Durham, North Carolina, USA; 8 Duke Clinical Research Institute, Duke University, Durham, North Carolina, USA

**Keywords:** African American, APOL1, proteinuria

## Abstract

**Introduction:**

Individuals of recent West African ancestry develop focal segmental glomerulosclerosis (FSGS) and hypertension-attributed end-stage kidney disease (HTN-ESKD) at 4 times the rate of White Americans. Two protein-coding variants of the Apolipoprotein L1 (APOL1) gene, G1 and G2, explain 50% to 70% of the excess risk of HTN-ESKD and FSGS among this group. Increased expression of G1 and G2 in the kidney, mediated by Janus kinase/signal transducer and activator of transcription (JAK-STAT) signaling, drive pathogenesis of these kidney diseases. Baricitinib is an orally active inhibitor of JAK1/2 that blocks APOL1 synthesis. The Janus kinase-STAT Inhibition to Reduce APOL1-Associated Kidney Disease (JUSTICE) trial is evaluating the antiproteinuric efficacy and safety of baricitinib in patients with APOL1-associated FSGS and HTN-attributed chronic kidney disease (HTN-CKD).

**Methods:**

JUSTICE is a single-center, randomized, double-blind, placebo-controlled, pilot phase 2 trial of baricitinib in patients with proteinuria, APOL1-associated FSGS or APOL1-associated HTN-CKD without diabetes. A total of 75 African American patients with APOL1-associated CKD, including 25 with FSGS and 50 with HTN-CKD, aged 18 to 70 years will be randomized 2:1 to daily treatment with baricitinib or placebo, respectively.

**Results:**

The primary efficacy end point will be percent change in urine albumin-to-creatinine ratio (UACR) from baseline to end of month 6. The primary safety end point will be incidence of clinically significant decreases in hemoglobin of ≥ 1g/dl.

**Conclusion:**

The phase 2 JUSTICE study will characterize the antiproteinuric efficacy and safety of JAK1/2 inhibition with baricitinib in patients with APOL1-associated FSGS and APOL1-associated HTN-CKD.

## Graphical abstract

**Figure:**
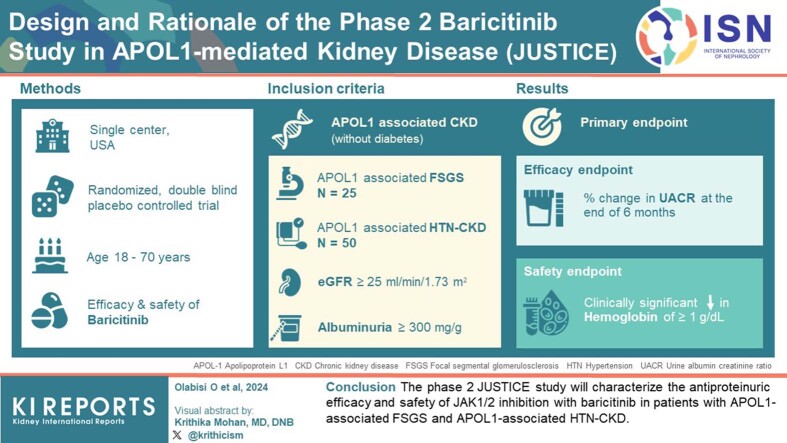

Among African Americans and other individuals of recent West African ancestry, FSGS is the leading cause of nondiabetic nephrotic syndrome, and HTN-CKD is the leading cause of ESKD not due to diabetes.^1^ Although social and structural determinants of health, including racism, poverty, discrimination, and lack of access to health care contribute to racial inequities in kidney health,^2^ 2 protein-coding variants of the gene apolipoprotein L1 (APOL1), G1 and G2, found exclusively in individuals of recent West African ancestry, account for much of the excess risk of FSGS and HTN-ESKD in African Americans.^3^ Approximately 13% of African Americans carry 2 risk variants of APOL1 (G1G1, G2G2, or G1G2), collectively referred to as high-risk genotypes (HRGs). By comparison, 70% of African Americans with FSGS and 40% with HTN-ESKD carry HRGs. Compared to African Americans with low-risk APOL1 genotypes (G0G0, G0G1, and G0G2), APOL1 HRGs increase the risk of FSGS 17-fold and HTN-ESKD up to 11-fold.^3^^,^^4^ Significant progress in understanding the pathomechanism of APOL1-mediated kidney disease (AMKD) has yielded promising therapeutic candidates; however, there is currently no FDA-approved drug for APOL1-associated kidney disease.^5^, ^6^, ^7^, ^8^ The use of nonspecific immunosuppressants for APOL1-associated FSGS and antihypertensives for APOL1-associated HTN-CKD fail to slow the rapid progression of these common forms of kidney disease to ESKD.^4^^,^^9^ There is an unmet need for specific therapy for AMKD.

Proteinuria and glomerular sclerosis, which are clinical and histopathologic consequences of sustained podocyte injury, are hallmarks of AMKD.^10^, ^11^, ^12^ Podocyte injury and subsequent proteinuria, with a 3-fold higher incidence in carriers of APOL1 HRG are the early clinical manifestations of AMKD.^11^^,^^12^ We and others have demonstrated that transgenic expression of APOL1 G1 or G2 in podocytes of zebrafish^13^ and mice^6^^,^^8^^,^^14^ is sufficient to cause podocyte injury, glomerular sclerosis, and proteinuria. Furthermore, inhibition of APOL1 expression by antisense oligonucleotides ameliorated podocyte injury and proteinuria in APOL1 transgenic mice.^14^ By comparison, expression of the reference APOL1 allele, G0, in mouse podocytes or tubular cells was nontoxic.^6^ Together, these results indicate that increased expression of APOL1 risk variants cause CKD via podocyte injury.

Observation that only 15% to 20% of carriers of HRG develop AMKD^15^ suggests that “second hits” are required to induce CKD in high-risk individuals. Two types of second hits have been identified as triggers of APOL1-mediated FSGS. First, elevated interferon states due to iatrogenic exposure,^16^ genetic mutations, such as TMEM173 (SAVI), which boosts synthesis and endogenous release of interferon,^17^ or disease processes such as systemic lupus which increase systemic interferons levels.^18^ Second, viral infections, especially HIV^4^^,^^19^ and most recently, COVID-19^20^ can cause collapsing glomerulopathy in African American carriers of 2 APOL1 risk variants. Experimental evidence suggests that COVID-19 causes collapsing FSGS by increasing systemic inflammatory cytokines, which in turn synergistically increase the expression of pathogenic APOL1 renal risk variants in podocytes and glomerular endothelial cells.^21^

JAK-STAT signaling is the common pathway that mediates the effect of interferons and noninterferon cytokines, including TNF-α, IL-6 and IL-1β, in inducing renal APOL1 expression.^21^^,^^22^ Inhibition of JAK1/2 effectively blocks APOL1 expression in human primary podocytes and glomerular endothelial cells.^21^ Importantly, our preliminary study shows that the JAK-STAT signaling pathway is potentiated in podocytes derived from patients with FSGS who carried HRG compared to podocytes from African Americans with HRG who are free of kidney disease.

The high population attributable risk of HTN-ESKD supports its causal link to pathogenic APOL1 variants;^15^ however, the second-hit trigger of APOL1-mediated pathogenesis of HTN-CKD is unknown. Induced expression of APOL1 risk variants in mice caused kidney failure without causing HTN.^8^ In patients with APOL1 HRG, antihypertensive medication did not slow the progression of HTN-CKD to ESKD.^9^ Together, this evidence suggests that HTN *per se* is less likely to be the second-hit trigger of APOL1-mediated HTN-CKD, but rather a consequence of it. We hypothesize that the inflammatory cytokines, including TNF-α, IL-6 and IL-1β, known to be elevated in patients with HTN, likely induce the expression of pathogenic APOL1 via JAK-STAT signaling, thereby contributing to the pathogenesis of APOL1-mediated, HTN-CKD.^23^, ^24^, ^25^, ^26^ Therefore, we hypothesize that clinically available inhibitors of JAK1/2 will block kidney APOL1 expression and thereby slow the progression of APOL1-mediated HTN-CKD.

JAK-STAT signaling is a ubiquitous pathway that contributes to erythropoiesis. Because baricitinib inhibits JAK2-mediated, erythropoietin-induced erythrocytosis, anemia is a potential side effect of baricitinib. However, data from 8 randomized clinical trials involving a total of 3492 participants and 7993 total person-years of exposure reported only a marginal reduction of −0.12 mmol/l in hemoglobin of patients receiving baricitinib.^27^ Moreover, previous trials in which baricitinib was used in patients with diabetic kidney disease did not show increased incidence of anemia or other hematologic abnormalities.^28^

The randomized, double-blind, placebo-controlled, pilot phase 2 JUSTICE trial has been initiated to study the efficacy and safety of baricitinib, a JAK1/2-specific inhibitor, in reducing proteinuria in patients with APOL1-associated FSGS or HTN-CKD over a period of 6 months. This paper describes the study design and methodology of the JUSTICE trial.

African Americans, who are overrepresented in advanced stage CKD and glomerulonephritis, are underrepresented in therapeutic trials in CKD accounting for 18% and 12%, respectively compared to 57% and 37% for White patients.^29^ Multiple barriers summarized in Table 1 account for this underrepresentation.^30^^,^^31^ To address these barriers, we established the Community APOL1 Research Engagement (CARE) registry (www.kidneycareandjustice.com) through which we engage, inform, and screen African Americans and other self-identified Black people in the United States of America for AMKD. By facilitating efficient identification of African Americans who are potentially eligible for the JUSTICE trial, the CARE registry will increase the enrolment of African Americans in this trial.

**Table 1:** Common barriers to participation in clinical trials and strategies employed by the CARE and JUSTICE studies aimed to reduce them

Key barriers to African American participation in clinical trials	Strategies for addressing key barriers in CARE and JUSTICE
1. Lack of kidney disease awareness.	Accessible CKD education and screening, free APOL1 genotyping.
2. Lack of awareness of therapeutic trial for kidney disease.	Engage and inform care providers and patients with clinical trial information.
3. The study site is a hassle to visit—it is far or requires too much time to access.	Use community-based research model. Meet participants where they are. Engage participants where they frequently congregate and feel most comfortable. Reimburse travel.
4. Implicit bias and inadequate cultural sensitivities.	Use culturally sensitive tools. Provide cultural sensitivity training for study staff.
5. Underrepresentation of African Americans and Black individuals among the research team.	The JUSTICE team reflects the target community: the principal investigator, coinvestigator, clinical research coordinator, and many of the study staff are African American or Black individuals.
6. Concern about potential health risk associated with participation in clinical trials.	Address the potential adverse effects of the investigational drug. Explain the rationale for a double-blind, placebo-controlled study design.
7. African Americans are not invited to participate in clinical trials due to the misconception that they are not interested.	Using culturally sensitive “just ask” approach, invite African Americans to participate in JUSTICE.
8. Distrust of medical research establishment due to history of exploitation and unethical research practices.	Acknowledge past problems. Describe safeguards to protect research participants. Invite and address participant’s concerns about medical research.
9. Discomfort with inherent uncertainty of trial participation.	Be transparent about potential personal health benefits and risks of participation.
10. Lack of access to a large population of potentially eligible African Americans.	Partner with African American community-based organizations, trusted influencers, and medical professionals that serve the African American communities.

## Methods

### Study Participants

Individuals aged 18 to 70 years, with biopsy-proven FSGS or clinically attributed HTN-associated CKD not due to diabetes, estimated glomerular filtration rate (eGFR) ≥25 ml/min per 1.73 m^2^ and albuminuria ≥300 mg/g, are eligible for the study if they carry APOL1 HRG. Inclusion and exclusion criteria are summarized in Tables 2 and 3. These individuals will be enrolled from clinics and the wider African American community through the Community APOL1 Research Engagement (CARE) registry (www.kidneycareandjustice.com). JUSTICE, as a part of the NEPTUNE observational study network,^32^ will also enroll participants from NEPTUNE. Individuals who self-report as Black or African American, Afro-Caribbean, Afro-Latino, or with recent African ancestry, between 18 to 70 years of age and residing in the United States are screened for history of high blood pressure, presence of albuminuria, reduced eGFR and presence of APOL1 HRG. It is possible that an individual with recent West African ancestry may be unaware of their geographical ancestry and may not self-identify as Black or African American. Therefore, if such individuals are known to carry APOL1 HRG, for instance through direct-to-consumer genetic testing, they will be eligible to screen for the JUSTICE trial, irrespective of racial or ethnic identity. It was recently reported that APOL1 G2 haplotype with a rare p.N264K single nucleotide polymorphism is protective against the risk of AMKD.^33^^,^^34^ Therefore, prospective participants with APOL1 genotypes of G1G2 or G2G2 with 1 or more p.N264K G2 haplotype will be ineligible to participate in JUSTICE. APOL1 genotype of participants entering JUSTICE is determined by using well-established Taqman based allelic discrimination or by Sanger sequencing.^21^

**Table 2:** JUSTICE key inclusion criteria

1. The patient is willing and able to provide signed informed consent.
2. The patient resides in the United States.
3. The patient is male or female aged 18–70 years.
4. The patient has biopsy-proven FSGS or clinically diagnosed HTN-CKD
5. The patient has estimated glomerular filtration rate (eGFR) ≥ 25 ml/min per1.73 m^2^ (based on CKD-EPI Cystatin C) at screening.
6. The patient has urine albumin-to-creatinine ratio (UACR) ≥ 300 mg/g at screening.
7. The patient has high-risk APOL1 genotype of G1G1, G1G2, G2G2.
8. Patients taking antihypertensive medications must be on a stable regimen for at least 1 month prior to enrollment.

**Table 3:** JUSTICE key exclusion criteria

1. The patient has diagnosis of diabetes mellitus.
2. The patient has biopsy-proven tip variant of FSGS.
3. The patient has history of kidney transplant.
4. The patient has sickle cell disease.
5. The patient has diagnosis of HIV or positive hepatitis B surface antigen.
6. The patient has significant liver disease with the most recent aspartate aminotransferase or alanine aminotransferase >1.5 times the upper limit of the normal range or the most recent available total bilirubin >1.5 times the upper limit of the normal range.
7. The patient has disqualifying laboratory abnormalities during screening, including hemoglobin <10 g/dl, absolute lymphocyte count <500 cells/mm^3^ or absolute neutrophil count < 1000 cells/mm^3^
8. The patient has systolic blood pressure ≥ 180 mm Hg or diastolic blood pressure ≥ 90 mm Hg.
9. The patient has current or prior history of treatment with a JAK Inhibitor.
10. The patient has active serious viral, bacteria, fungal or parasitic infection.
11. The patient has symptomatic herpes zoster infection within 12 weeks prior to study entry.
12. The patient has active malignancy.
13. The patient is pregnant, breastfeeding, or plans to become pregnant during the study period.
14. The patient, in the opinion of the investigator, is unable to comply with the requirements of the study.

FSGS, focal segmental glomerulosclerosis; JAK, Janus kinase.

### Sample Size and Power Calculation

In Table 4, we present sample size estimates, assuming 30%, 35%, and 40% differences between baricitinib and placebo, power of 80% or 85%, different SDs for percent change in UACR from baseline to the end of month 6, and the 2:1 active:placebo randomization scheme. For the purpose of the power calculation, we assumed 0% change in UACR in the placebo arm. Assuming the higher SD of 50%, a sample of 75 participants (50 in treatment arm and 25 in placebo arm) will provide 80% power to detect a 35% decrease in UACR at month 6 compared to placebo in the overall study population with a 2-sided significance level of 5%. A total of 25 patients with FSGS and 50 patients with HTN-associated CKD will be enrolled into the study. There will be no planned interim analyses although the Data Safety and Monitoring Board will have access to unblinded results at their regularly scheduled meetings. Approval of the study design and procedures was obtained from the Duke University Institutional Review Board before enrollment. Written informed consent is obtained for participation in the study.

**Table 4:** Sample size and power calculation

Standard deviation	Difference	Power	Required Sample size		
			Total	Baricitinib	Placebo
SD = 0.33	0.30	80%	45	30	15
		85%	51	34	17
	0.35	80%	36	24	12
		85%	39	26	13
	0.4	80%	27	18	9
		85%	30	20	10
SD = 0.50	0.30	80%	102	68	34
		85%	117	78	39
	0.35	80%	75	50	25
		85%	87	58	29
	0.4	80%	60	40	20
		85%	66	44	22

### Randomization and Intervention

**Figure 1:**
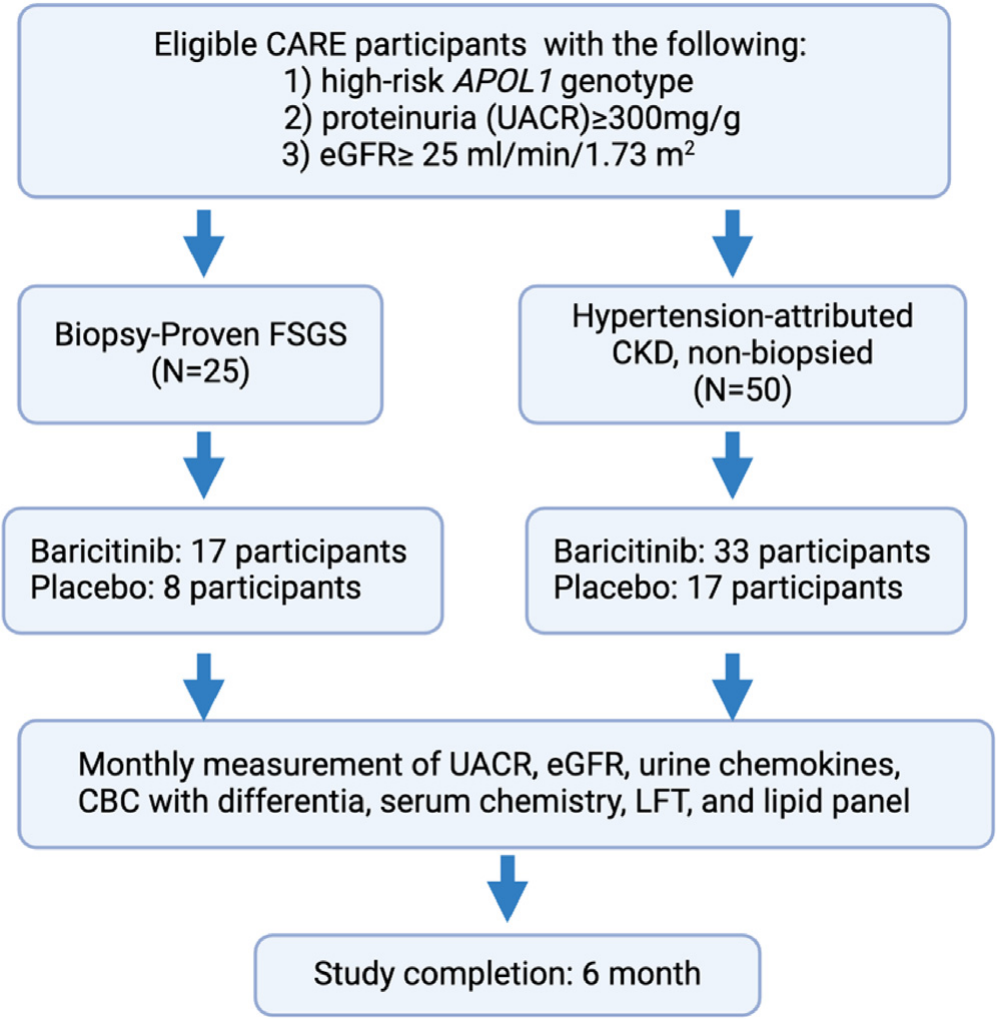
Flow of participants from CARE to JUSTICE. Through Community APOL1 Research Engagement (CARE) registry, community-dwelling individuals will be screened for carriage of high-risk APOL1 genotype, the presence of proteinuria, and eGFR will be measured in those with UACR ≥ 300 mg/g. Similar screening will be performed on patients with known kidney disease (FSGS or CKD without diabetes) in clinical settings. CARE participants (FSG2, *n* = 25 and hypertension-attributed CKD, *n* = 50) who have high-risk APOL1 genotype proteinuria (UACR ≥ 300 mg/g) and eGFR ≥ 25 ml/min per 1.7m^2^ will be consented for enrolment into JUSTICE trial. CBC, complete blood count; CKD, chronic kidney disease; eGFR, estimated glomerular filtration rate; FSGS, focal segmental glomerulosclerosis; LFT, liver function test; UACR, urine albumin-to-: creatinine ratio.

The study design is illustrated in Figure 1. Participants will be required to be on standard-of-care, including a stable dose of angiotensin-converting enzyme inhibitors or angiotensin 2 receptor blocker for at least 30 days before randomization. Participants must have systolic blood pressure less than 180 mm Hg or diastolic blood pressure less than 90 mm Hg prior to randomization. Duke Pharmacy Investigational Drug Services will randomly assign participants 2:1 to baricitinib or placebo. According to this schema, 17 participants with FSGS will be randomized to baricitinib and 8 to placebo. Among patients with HTN-CKD, 33 will be randomized to baricitinib and 17 to placebo. We opted for this 2:1 randomization to maximize the number of patients treated with baricitinib who will support within group analyses of change in UACR over time. Baricitinib will be dose-adjusted for eGFR: 1 mg once daily orally for eGFR of 30 to 50 ml/min per 1.73 m^2^; 2 mg once daily orally for eGFR > 50 ml/min per 1.73 m^2^. The duration of treatment will be 6 months.

### Data Collection

**Figure 2:**
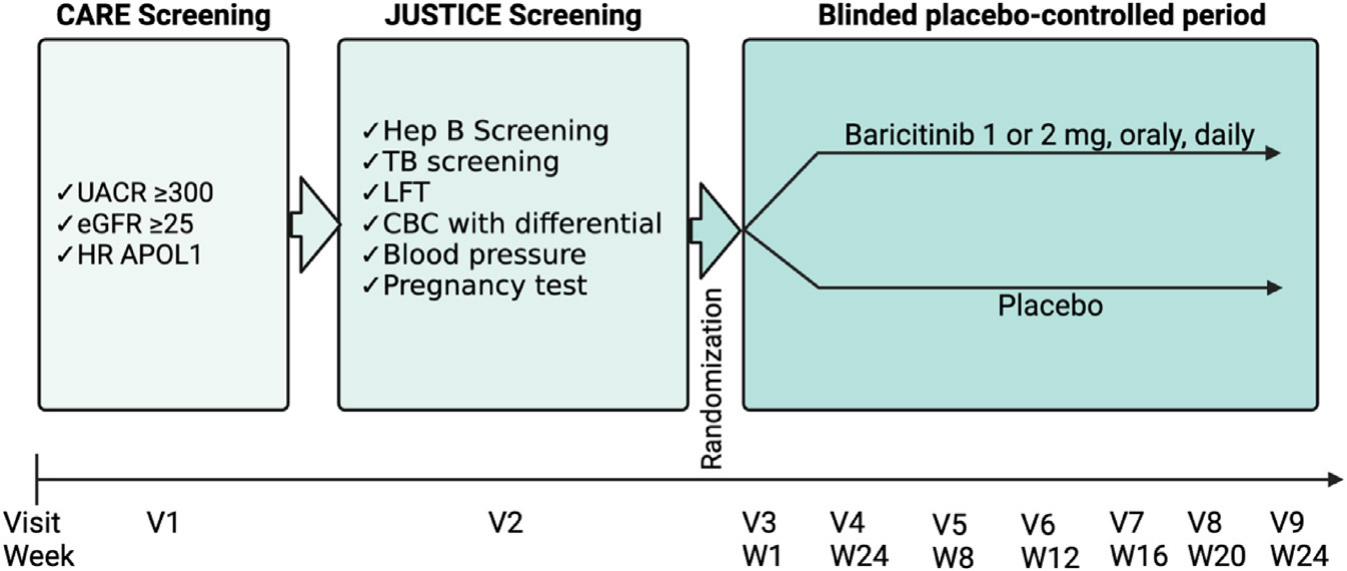
JUSTICE Study Design. A single-center, randomized, double-blind, placebo-controlled, pilot phase 2 trial of baricitinib in patients with proteinuria, APOL1-associated FSGS or APOL1-associated, hypertension-attributed CKD without diabetes. Following CARE screening (Visit 1), JUSTICE screening (visit 2) requires in-person visit and includes collection of blood sample. Participants will have the option of virtual visits for study visits 3 to 9.

We will collect the following baseline data: age, biological sex, height, weight, blood pressure, eGFR, UACR, past medical and family history, current medications, previous treatments for CKD, and duration of CKD, when known, including dates of and results of kidney biopsies. The baseline and monthly eGFR will be determined with the CKD-Epidemiology Collaboration Cystatin C equation.^35^ The baseline and monthly UACR values will be the average of 2 UACR measurements based on 2 first morning voids separated by at least 2 days. Furthermore, at each monthly study visit, blood samples will be collected to measure liver function tests, serum chemistries, and complete blood counts. At each study visit when new 30-day supplies of medications will be provided, we will assess adherence by performing pill counts for the previous months and collect urine to be stored for future measurement of urinary chemokines. We will also collect data on any adverse effects. The CARE screening (visit 1) and JUSTICE screening (visit 2) visits will be in-person (Figure 2). However, participants will have the option of virtual visits for study visits 3 to 9.

### End Points

The primary efficacy end point is percent change in UACR from baseline to end of month 6. Because anemia is a relevant potential adverse effect of baricitinib for which patients with CKD may be more vulnerable, the primary safety end point is incidence of a clinically significant decrease in hemoglobin of ≥1 g/dl. We will measure UACR at baseline and at the end of each of the 6 months for a total of 7 assessments. We will also assess eGFR at baseline and monthly as a secondary safety measure. Because of the known effect of baricitinib to reduce tubular secretion of creatinine^28^ we will measure eGFR using the CKD-Epidemiology Collaboration Cystatin C equation^35^ rather than a creatinine-based equation. As an exploratory analysis of target engagement by baricitinib, we will also measure the chemokine biomarker, urine CXCL9-11, with standard assays at baseline and at each study visit.

### Statistical Analyses

We will use standard descriptive statistics to compare clinical characteristics at baseline according to the treatment arm. The primary analysis will be a comparison of differences in percent change in UACR from baseline to month 6 across treatment arms in the overall study population using 2-sample t-tests. This primary analysis will be conducted according to the intention-to-treat principle with participants analyzed according to the treatment arm to which they were randomized, regardless of subsequent crossover or post-randomization treatment. In secondary analyses, we will compare the effects of baricitinib versus placebo on percent change in UACR within each of the APOL1 disease strata (FSGS and HTN) separately. In further analyses, we will assess the between-group and within-group changes in UACR using linear mixed models with visit number (baseline, months 1–6) representing the repeated-measures factor and randomization arm treated as a fixed-effect term. We will select the appropriate covariance matrix (e.g., compound symmetry, autoregressive, unstructured, etc.) based on the data to account for repeated observations. The statistical test for significant differences between the groups will be the time x group interaction that assesses the overall differences between groups in longitudinal UACR. The mixed model analyses will enable us to assess within subject changes in UACR over time, and to adjust for any imbalances in baseline factors that may occur in this relatively small study. We will use the same strategy involving linear mixed models to assess the effects of the intervention on eGFR and the chemokine biomarkers (CXCL9–11). For the primary assessment of safety, we will investigate the incidence of hemoglobin reduction by ≥1 g/dl in each arm and the mean change in hemoglobin from baseline to the end of study in each arm. We will also report adherence based on pill count data and use standardized methods to report all adverse events.

## Discussion

Although African Americans with high-risk APOL1 genotypes have the highest risk of developing idiopathic FSGS and HTN-attributed ESKD,^3^^,^^4^^,^^9^ they are underrepresented in clinical trials of therapeutic agents.^36^^,^^37^ Therefore, there is an urgent unmet need for novel therapies for AMKD. The JUSTICE study is designed to address this unmet need by testing the antiproteinuric efficacy and safety of baricitinib in individuals with AMKD. Preclinical studies demonstrate that the JAK-STAT signaling pathway is the key upstream inducer of APOL1 expression in podocytes and glomerular endothelial cells.^21^^,^^22^ In mouse models, expression of G1 or G2 APOL1 variants in podocytes causes podocyte injury, proteinuria, and kidney injury.^6^^,^^8^^,^^38^ Baricitinib inhibits JAK1/2 and thereby blocks APOL1 expression in podocytes and other kidney cells. JUSTICE is designed to test whether the inhibition of renal APOL1 expression by baricitinib would reduce APOL1-mediated podocyte injury as manifested by proteinuria.

To assess the effect of baricitinib on halting APOL1-induced podocytopathy and glomerulopathy, JUSTICE will compare UACR at the end of 6 month of baricitinib or placebo with baseline proteinuria. We predict that inhibiting expression of pathogenic APOL1 proteins in podocytes will attenuate APOL1-mediated podocyte injury and thereby improve UACR. The use of changes in proteinuria rather than changes in glomerular filtration rate as the primary end point of JUSTICE is due to practical considerations. Treatment-induced improvement in proteinuria is detectable within 6 months. In contrast, longer durations are required for significant changes in glomerular filtration rate to emerge. Nonetheless, changes in eGFR will be measured as an exploratory end point in JUSTICE. The well-established role of podocyte injury and the associated proteinuria as early and frequent phenotypes of AMKD further justifies the use of proteinuria reduction as the primary end point. Detection of a clinically meaningful proteinuria reduction by baricitinib in JUSTICE would provide a strong basis for a longer phase 3 clinical trial in which changes in kidney function would be the primary end point.

It would be ideal to make the stable use of sodium-glucose cotransporter-2-inhibitors (SGLT-2is) at baseline a precondition for enrolment because SGLT-2is reduce kidney disease progression and mortality in patients with CKD, regardless of diabetes status.^39^ However, a recent cross-sectional registry analysis of 49,587 nondiabetic patients with CKD in a large integrated health care system in the US, reported that only a paltry 0.3% of these patients were prescribed SGLT-2i.^40^ Therefore, the current low rate of SGLT-2i prescription in the nondiabetic CKD population would constitute a significant, additional barrier to enrolment of otherwise eligible patients into the JUSTICE trial. Moreover, it is not known that SGLT-2is specifically reduce the rate of progression of AMKD. For these reasons, we did not require the stable use of SGLT-2i as a precondition for enrolment in JUSTICE trial.

To assess the safety of baricitinib in patients with AMKD, we will compare the incidence of anemia a potential adverse effect of JAK-inhibitors in the baricitinib versus placebo recipients. Previous trials in which baricitinib was used in patients with diabetic kidney disease did not show increased incidence of anemia, leucopenia, or thrombocytopenia in participants who received baricitinib versus placebo.^28^ Based on this precedent, our expectation is that baricitinib will also be well-tolerated in JUSTICE.

A unique feature of JUSTICE is the active engagement of African Americans/Black individuals both in the community and in clinical settings for study recruitment. A similar community-engaging strategy is being effectively employed by the ongoing National Institutes of Health–funded APOL1 Long-term Kidney Transplantation Outcomes Network (APOLLO) (NCT03615235).^41^ Through the CARE registry, JUSTICE investigators will work with a well-established network of regional and national community partners who are trusted leaders, connectors, and influencers in the broader African American community. These partners are committed to advancing health equity and addressing racial health disparities. This includes leadership of religious, educational, civic, and social organizations. The CARE registry provides accessible information about the high burden of CKD, especially ESKD, and the causal role of APOL1 HRGs. Test results are returned to all participants who express desire to receive them. Genetic counseling is offered to interested participants. This bidirectional community engagement and screening activity will help to increase awareness and access to trustworthy kidney disease information, screening, and clinical research with the goal of reducing the longstanding trust deficit between the African American community and biomedical research organizations. It also increases the opportunity for identifying individuals with APOL1 HRG who have proteinuric kidney disease, not due to diabetes, that could be further screened for participation in JUSTICE study.

The combined strategy of community engagement and mechanism-informed therapeutic trial could serve as a model for future trials aimed at African Americans and other ethnic groups who are underrepresented in clinical trials.

## Disclosure

OAO is an advisor to MazeTherapeutics and Podium Bio; and received research support from Icagen and Eli Lilly. All the other authors declared no competing interests.
